# Conscious sedation compared to general anesthesia for intracranial mechanical thrombectomy: A meta‐analysis

**DOI:** 10.1002/brb3.2161

**Published:** 2021-05-07

**Authors:** Huasu Shen, Xiaoyu Ma, Zhen Wu, Xian Shao, Jingjing Cui, Bao Zhang, Mohamed EA Abdelrahim, Jin Zhang

**Affiliations:** ^1^ Department of Anesthesiology The Fourth Hospital of Shijiazhuang Shijiazhuang China; ^2^ Department of Anesthesiology The Fourth Hospital of Hebei Medical University Shijiazhuang China; ^3^ Department of Anesthesiology Cangzhou Hospital of Integrated TCM‐WM·Hebei Cangzhou China; ^4^ Clinical Pharmacy Department Faculty of Pharmacy Beni‐Suef University Beni‐Suef Egypt

**Keywords:** acute ischemic stroke, anesthetic strategy, conscious sedation, endovascular therapy, general anesthesia

## Abstract

**Introduction:**

Endovascular therapy is the standard of care for severe acute ischemic stroke caused by large‐vessel occlusion in the anterior circulation, but there is a debate on the optimal anesthetic approach during this therapy. Meta‐analyses of observational studies suggest that general anesthesia increases disability and death compared with conscious sedation However, their results are conflicting. This meta‐analysis study was performed to assess the relationship between the effects of general anesthesia compared to conscious sedation during endovascular therapy for acute ischemic stroke.

**Methods:**

Through a systematic literature search up to August 2020, 18 studies included 4,802 subjects at baseline with endovascular therapy for acute ischemic stroke and reported a total of 1,711 subjects using general anesthesia and 1,961 subjects using conscious sedation were found. They recorded relationships between the effects of general anesthesia compared to conscious sedation during endovascular therapy for acute ischemic stroke. Odds ratio (OR) or Mean differences (MD) with 95% confidence intervals (CIs) were calculated between the effect of general anesthesia compared to conscious sedation during endovascular therapy for acute ischemic stroke using the dichotomous or contentious methods with a random or fixed‐effect model.

**Results:**

No significant difference were found between general anesthesia and conscious sedation during the endovascular therapy for acute ischemic stroke in functional independence at 90 days (OR, 0.78; 95% CI, 0.44–1.40, *p* = 40); successful recanalization at 24 hr (OR, 1.23; 95% CI, 0.62–2.41, *p* = 55); mortality at 90 days (OR, 1.36; 95% CI, 0.83–2.24, *p* = .22); interventional complication (OR, 1.24; 95% CI, 0.76–2.02, *p* = .40); symptomatic intracranial hemorrhage (OR, 0.64; 95% CI, 0.41–0.99, *p* = .05); aspiration pneumonia (OR, 0.96; 95% CI, 0.58–1.58, *p* = .87); and National Institute of Health Stroke Scale score after 24 hr (MD, 0.38; 95% CI, −1.15–1.91, *p* = .62); with relative relationship favoring general anesthesia only in decreasing the symptomatic intracranial hemorrhage.

**Conclusions:**

General anesthesia has no independent relationship compared to conscious sedation during the endovascular therapy for acute ischemic stroke with a relative relationship favoring general anesthesia only in decreasing the symptomatic intracranial hemorrhage.

This relationship encouraged us to recommend either anesthetic strategy during the endovascular therapy for acute ischemic stroke with no possible fear of higher complication.

## Graphical abstract

What's already known about this topic?Endovascular therapy is the standard of care for severe acute ischemic stroke caused by large‐vessel occlusion in the anterior circulation, but there is a debate on the optimal anesthetic approach during this therapy. Meta‐analyses of observational studies suggest that general anesthesia increases disability and death compared with conscious sedation However, their results are conflicting. This meta‐analysis study was performed to assess the relationship between the effects of general anesthesia compared to conscious sedation during endovascular therapy for acute ischemic stroke.What does this article add?General anesthesia has no independent relationship compared to conscious sedation during the endovascular therapy for acute ischemic stroke with a relative relationship favoring general anesthesia only in decreasing the symptomatic intracranial hemorrhage.This relationship encouraged us to recommend either anesthetic strategy during the endovascular therapy for acute ischemic stroke with no possible fear of higher complication.

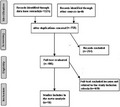

## 1 INTRODUCTION

Acute ischemic stroke is one of the main causes of mortality and disability worldwide. Endovascular therapy is a standard of care treatment for severe acute ischemic stroke produced by large‐vessel occlusion, suggested by international guidelines. (Touma et al., 2016) When performing endovascular therapy, there are two anesthetic types commonly used to make the acute ischemic stroke subject immovable. They are general anesthesia and conscious sedation. Which anesthetic type could result in the best outcomes is still an open question. General anesthesia with intubation might be related to less pain and movement and lower aspiration risk. (Emiru et al., 2014) Conscious sedation with the spontaneously breathing subject may be related to less time and hemodynamic instability and lower ventilation‐related problems risk.

Former systematic review and meta‐analyses (Abou‐Chebl et al., 2010; Brinjikji et al., 2015; Wan et al., 2019) and observational studies (Abou‐Chebl et al., 2010, 2015; Just et al., 2016; Mundiyanapurath et al., 2015) showed worse results from general anesthesia compared with conscious sedation in endovascular therapy. Though, most of the studies might have selection bias since subjects with more serious strokes are mostly treated under general anesthesia. (Abou‐Chebl et al., 2010,2015; Berkhemer et al., 2016; Bracard et al., 2016; Brinjikji et al., 2015; Just et al., 2016; Mundiyanapurath et al., 2015; Nichols et al., 2018) Also, randomized clinical trials mostly have a low sample size to identify significant differences in primary results. (Arthur et al., 2019; Löwhagen Hendén et al., 2017; Schönenberger et al., 2016; Simonsen et al., 2018) Those meta‐analysis studies had very big limitations which are the small sample size. So, a new and meta‐analysis study is warranted. A recent meta‐analysis including only 3 randomized clinical trials with a large sample size reported that general anesthesia delivers favorable functional independence during endovascular treatment for acute ischemic stroke, compared to conscious sedation. (Zhang et al., 2019).

Bearing in mind the difference and the limitation of former meta‐analysis studies, the present meta‐analysis aimed to compare the effect of general anesthesia to that of conscious sedation on subjects with ischemic stroke undertaking endovascular therapy.

## 2 METHODS

The study performed here followed the meta‐analysis of studies in the epidemiology statement, (Stroup et al., 2000) which was conducted following an established protocol.

### 2.1 Study selection

Randomized controlled trials included were that reported statistical measures of relationship (odds ratio [OR], incidence rate ratio or relative risk, with 95% confidence intervals [CIs]) between the effect of general anesthesia compared to conscious sedation during endovascular therapy for acute ischemic stroke. All types of participant populations were ≥18 years old.

**FIGURE 1:**
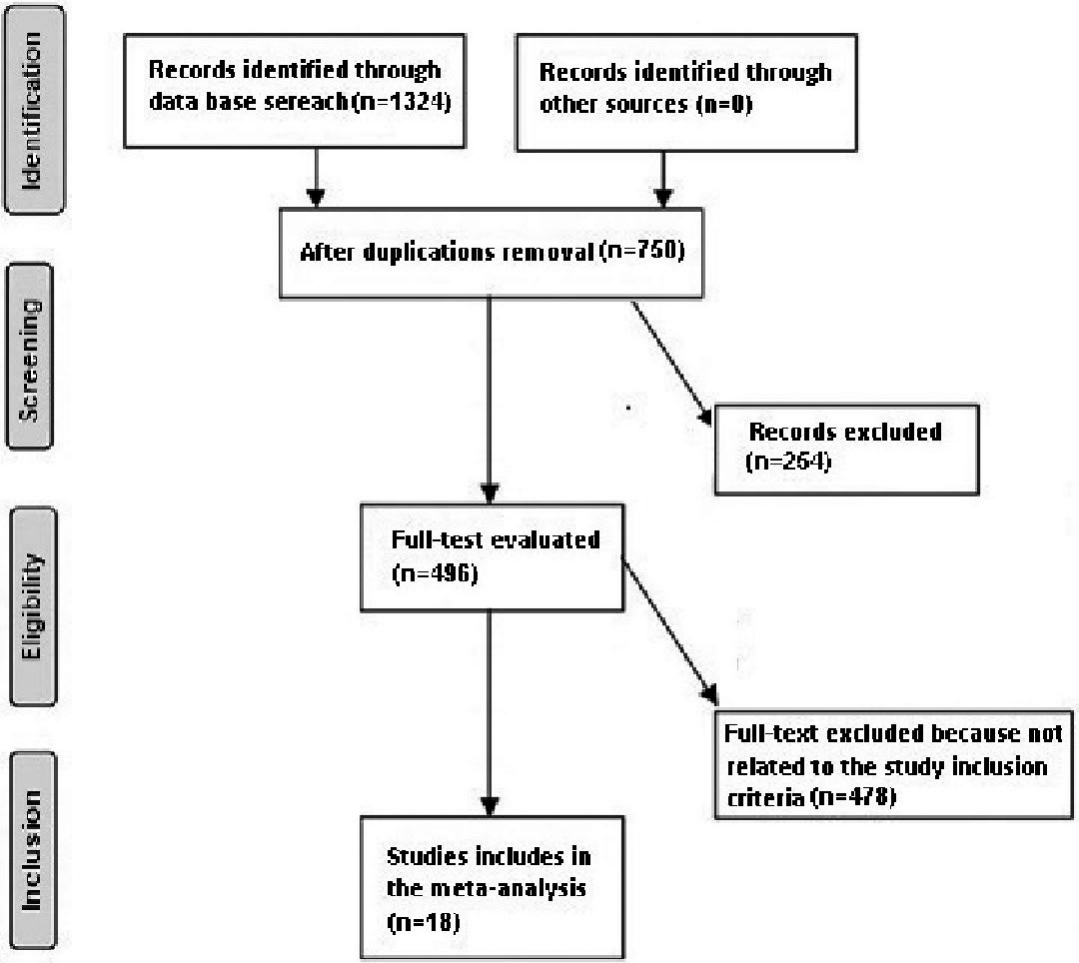
Schematic diagram of the study procedure

Only human studies in any language were considered. Inclusion was not restricted by study size or publication type. Publications excluded were studies that did not provide a measure of a relationship. Figure 1 shows the whole study procedure.

Articles were integrated into the meta‐analysis when the following inclusion criteria were met:

1. The study was randomized clinical trials.
2. The target population is subjects with ischemic stroke undertaking endovascular therapy.
3. The intervention program was based on general anesthesia and conscious sedation.
4. The study included a comparison between general anesthesia and conscious sedation

### 2.2 Identification

A protocol of search strategies was prepared according to the PICOS principle, and we defined it as follow: P (population): subjects with ischemic stroke undertaking endovascular therapy; I (intervention/exposure): general anesthesia and conscious sedation; C (comparison): general anesthesia compared to conscious sedation; O (outcome): functional independence at 90 days, successful recanalization at 24 hr, mortality at 90 days, interventional complication, symptomatic intracranial hemorrhage, aspiration pneumonia, National Institute of Health Stroke Scale score after 24 hr, and symptomatic intracranial hemorrhage; and S (study design): no restriction.

First, we conducted a systematic search of OVID, Embase, Cochrane Library, PubMed, and Google scholar till June 2020, using a combination of keywords and similar words for anesthetic strategy, conscious sedation, general anesthesia, endovascular therapy, and acute ischemic stroke as shown in Table 1. All identified studies were combined in an EndNote file, duplicates were discarded, and the title and abstracts were reviewed to exclude studies that did not report a relationship between the effect of general anesthesia compared to conscious sedation during endovascular therapy for acute ischemic stroke, based on the previously mentioned inclusion and exclusion criteria. The remaining articles were examined for correlated information.

**TABLE 1:** Search Strategy for Each Database

Database	Search strategy
Pubmed	#1 "Anesthetic strategy"[MeSH Terms] OR "Conscious sedation"[All Fields] OR "General anesthesia"[All Fields] #2 "endovascular therapy"[MeSH Terms] OR "Anesthetic strategy" OR "endovascular therapy"[All Fields] #3 #1 AND #2
Embase	'Anesthetic strategy'/exp OR 'Conscious sedation'/exp OR 'General anesthesia' #2 'endovascular therapy'/exp OR 'endovascular therapy' #3 #1 AND #2
Cochrane library	(Anesthetic strategy):ti,ab,kw (Conscious sedation):ti,ab,kw OR (General anesthesia) :ti,ab,kw (Word variations have been searched) #2 (endovascular therapy): ti,ab,kw (endovascular therapy) #3 #1 AND #2

### 2.3 Screening

Data were abridged based on study‐associated and subject‐associated features onto a consistent form. the last name of the primary author, period of study, year of publication, country, region of the studies, and study design; population type, the total number and the number of subjects used conscious sedation, or general anesthesia, demographic data, and clinical and treatment characteristics; and method of assessment; result assessment; and statistical analysis OR or relative risk, along with 95% CI, of the relationship and its result. (Gupta et al., 2018) If a study qualified for inclusion based upon the aforementioned principles, data were extracted independently by two authors (Huasu Shen, and Xiaoyu Ma). In case of disagreement, agreement, Jin Zhang provided a final option. When there were different data from 1 study, we extracted them separately. The risk of bias in these studies; individual studies were evaluated using the quality in prognosis studies tool, which evaluates validity and bias in studies of prognostic factors across six domains: participation, attrition, prognostic factor measurement, confounding measurement, and account, outcome measurement, and analysis and reporting. (Hayden et al., 2013) Any inconsistencies were addressed by a re‐evaluation of the original article.

### 2.4 Eligibility

The primary result concentrated on the effect of general anesthesia compared to conscious sedation during endovascular therapy for acute ischemic stroke. A comparison between the effect of conscious sedation and general anesthesia was extracted to form a summary.

### 2.5 Inclusion

Sensitivity analyses were limited only to studies reporting the relationship between the effects of general anesthesia compared to conscious sedation during endovascular therapy for acute ischemic stroke. For subgroup and sensitivity analysis, we used comparisons between conscious sedation and general anesthesia as references.

### 2.6 Statistical analysis

The dichotomous or contentious methods with a random or fixed‐effect model were used to calculate odds ratio (OR) or mean differences (MD) and 95% CI. We calculated the *I* ^2^ index; the *I* ^2^ index is between 0% and 100%. Values of approximately 0%, 25%, 50%, and 75% indicate no, low, moderate, and high heterogeneity, respectively. (Sheikhbahaei et al., 2016) When *I* ^2^ was higher than 50%, we chose the random effect model; when it was lower than 50%, we used the fixed‐effect model. A subgroup analysis was performed by stratifying the original evaluation per outcome categories as described before. In this analysis, a *p*‐value for differences between subgroups of < 0.05 was considered statistically significant. Publication bias was evaluated quantitatively using the Egger regression test (publication bias considered present if *p* ≥ .05), and qualitatively, by visual examination of funnel plots of the logarithm of ORs or MDs versus their standard error (*SE*). (Higgins, 2003) All *p*‐values were two tailed. All calculations and graphs were performed using reviewer manager version 5.3 (The Nordic Cochrane Centre, The Cochrane Collaboration, Copenhagen, Denmark).

## 3 RESULTS

A total of 1,324 unique studies were identified, of which 18 studies, from 2010 until 2020 in humans, satisfied the inclusion criteria and were included in the study. (Abou‐Chebl et al., 2010, 2015; Berkhemer et al., 2016; Davis et al., 2012; Goldhoorn et al., 2020; Hassan et al., 2012; Jumaa et al., 2010; Just et al., 2016; Langner et al., 2013; Li et al., 2014; Löwhagen Hendén et al., 2017; Mundiyanapurath et al., 2015; Nichols et al., 2018; Ren et al., 2020; Schönenberger et al., 2016; Simonsen et al., 2018; Sørensen et al.,^2019^; Zussman et al., 2018) The details of the included studies are listed in Table 2.

**TABLE 2:** Characteristics of the selected studies for the meta‐analysis

Study	Country	Total	General anesthesia	Conscious sedation
Abou‐Chebl et al. (2010)	USA	980	426	554
Jumaa et al. (2010)	USA	126	53	73
Davis et al. (2012)	Canada	129	48	48
Hassan et al. (2012)	USA	136	53	83
Langner et al. (2013)	Germany	124	19	105
Li et al. (2014)	USA	109	35	74
Abou‐Chebl et al. (2015)	USA	434	147	269
Mundiyanapurath et al. (2015)	Germany	44	29	15
Berkhemer et al. (2016)	Netherlands	216	79	137
Just et al. (2016)	Canada	109	42	67
Schönenberger et al. (2016)	Germany	150	73	77
Löwhagen Hendén et al. (2017)	Sweden	321	54	45
Nichols et al. (2018)	USA	75	49	26
Simonsen et al. (2018)	USA	128	65	63
Zussman et al. (2018)	USA	91	46	45
Sørensen et al. (2019)	Denmark	128	64	64
Goldhoorn et al. (2020)	Netherlands	1,376	381	174
Ren et al. (2020)	China	126	48	42
Total		**4,802**	**1711**	**1961**

The 18 studies included 4,802 subjects at baseline with endovascular therapy for acute ischemic stroke and reported a total of 1,711 subjects using general anesthesia and 1,961 subjects using conscious sedation. Those studies had subjects using general anesthesia compared to conscious sedation during endovascular therapy for acute ischemic stroke. 11 studies reported data stratified by the anesthetic strategy related to the functional independence (modified Rankin Scale scores of ≤2) at 90 days; six studies reported data stratified by the anesthetic strategy related to the successful recanalization (modified Thrombolysis in Cerebral Infarction 2b‐3) at 24 hr; 12 studies reported data stratified by the anesthetic strategy related to the mortality at 90 days; five studies reported data stratified by the anesthetic strategy related to the interventional complication; seven studies reported data stratified by the anesthetic strategy related to the symptomatic intracranial hemorrhage; 10 studies reported data stratified by the anesthetic strategy related to the aspiration pneumonia; seven studies reported data stratified by the anesthetic strategy related to the National Institute of Health Stroke Scale score after 24 hr. The significant impact of general anesthesia compared to conscious sedation during endovascular therapy for acute ischemic stroke was not observed in all the parameters studied with relatively lower symptomatic intracranial hemorrhage in subjects using general anesthesia.

The study size ranged from 44 to 1,376 subjects at the start of the study with subjects using general anesthesia ranged from 19 to 426, and subjects using conscious sedation ranged from 15 to 554.

**FIGURE 2:**
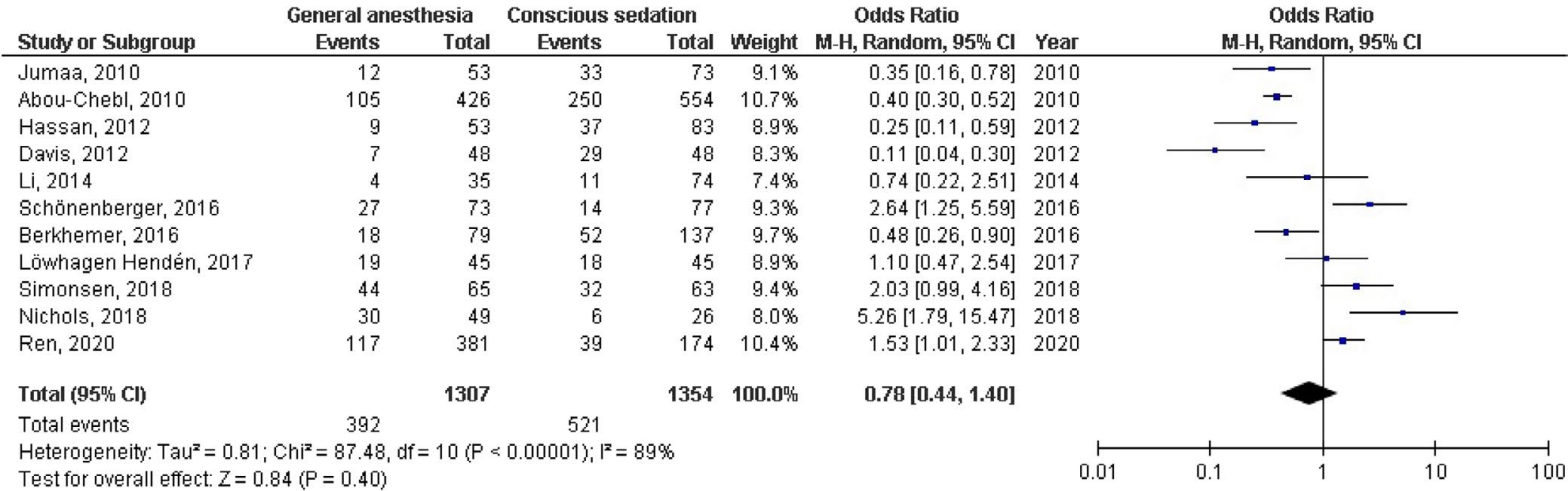
Forest plot of the general anesthesia versus conscious sedation during the endovascular therapy for acute ischemic stroke related to functional independence at 90 days

**FIGURE 3:**
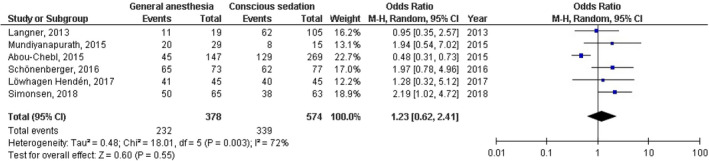
Forest plot of the general anesthesia versus conscious sedation during the endovascular therapy for acute ischemic stroke related to successful recanalization at 24 hr

**FIGURE 4:**
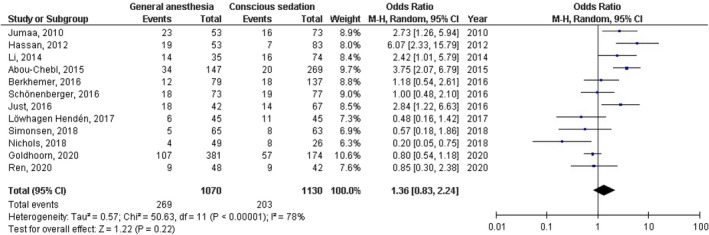
Forest plot of the general anesthesia versus conscious sedation during the endovascular therapy for acute ischemic stroke related to mortality at 90 days

**FIGURE 5:**
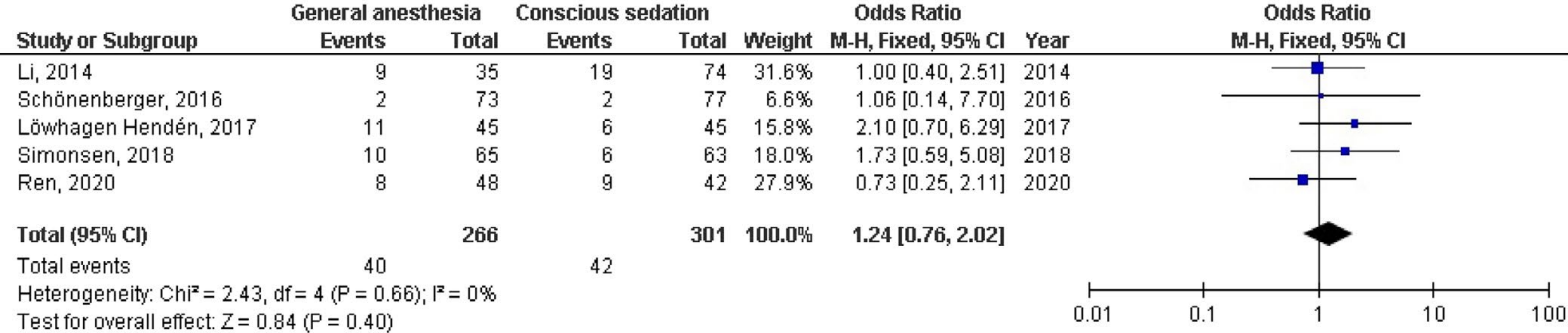
Forest plot of the general anesthesia versus conscious sedation during the endovascular therapy for acute ischemic stroke related to interventional complication

**FIGURE 6:**
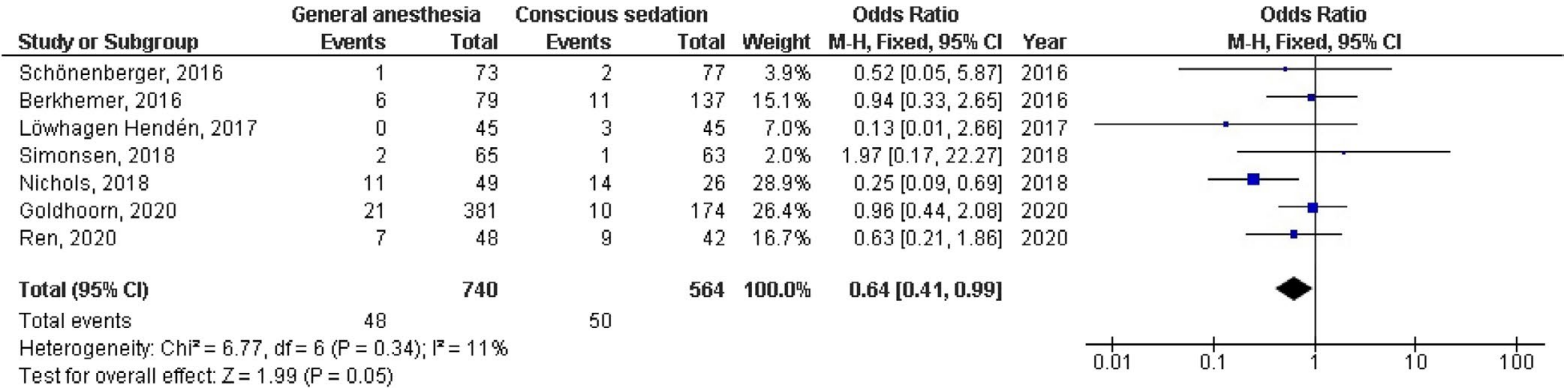
Forest plot of the general anesthesia versus conscious sedation during the endovascular therapy for acute ischemic stroke related to symptomatic intracranial hemorrhage

**FIGURE 7:**
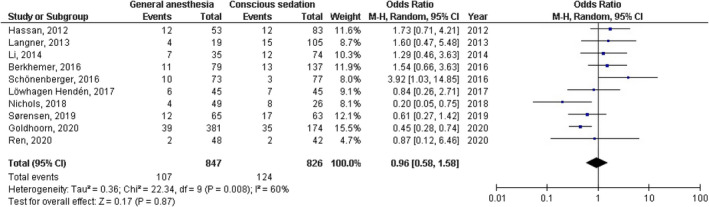
Forest plot of the general anesthesia versus conscious sedation during the endovascular therapy for acute ischemic stroke related to aspiration pneumonia

**FIGURE 8:**
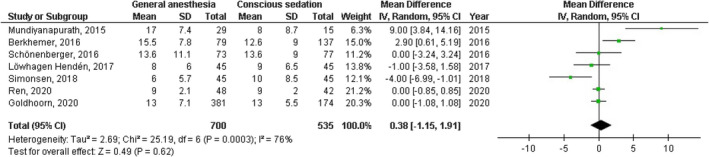
Forest plot of the general anesthesia versus conscious sedation during the endovascular therapy for acute ischemic stroke related to National Institute of Health Stroke Scale score after 24 hr

No significant difference was found between general anesthesia and conscious sedation during the endovascular therapy for acute ischemic stroke in functional independence (modified Rankin Scale scores of ≤2) at 90 days (OR, 0.78; 95% CI, 0.44–1.40, *p* = 40) with high heterogeneity (*I* ^2^ = 89%); successful recanalization (modified Thrombolysis in Cerebral Infarction 2b‐3) at 24 hr (OR, 1.23; 95% CI, 0.62–2.41, *p* = 55) with moderate heterogeneity (*I* ^2^ = 72%); Mortality at 90 days (OR, 1.36; 95% CI, 0.83–2.24, *p* = .22) with high heterogeneity (*I* ^2^ = 78%); interventional complication (OR, 1.24; 95% CI, 0.76–2.02, *p* = .40) with no heterogeneity (*I* ^2^ = 0%); Symptomatic intracranial hemorrhage (OR, 0.64; 95% CI, 0.41–0.99, *p* = .05) with no heterogeneity (*I* ^2^ = 11%); aspiration pneumonia (OR, 0.96; 95% CI, 0.58–1.58, *p* = .87) with moderate heterogeneity (*I* ^2^ = 60%); and National Institute of Health Stroke Scale score after 24 hr (MD, 0.38; 95% CI, −1.15–1.91, *p* = .62) with high heterogeneity (*I* ^2^ = 76%) as shown in Figures 2, 3, 4, 5, 6, 7, 8.

A stratified analysis of studies that did and did not adjust for age and gender was not performed because not enough studies reported were adjusted for this factor.

Based on the visual inspection of the funnel plot as well as on quantitative measurement using the Egger regression test, there was no evidence of publication bias (*p* = .87).

## 4 DISCUSSION

The 18 studies included 4,802 subjects at baseline with endovascular therapy for acute ischemic stroke and reported a total of 1,711 subjects using general anesthesia and 1,961 subjects using conscious sedation. (Abou‐Chebl, 2010, 2015; Berkhemer, 2016; Davis, 2012; Goldhoorn, 2020; Hassan, 2012; Jumaa, 2010; Just, 2016; Langner,; Li, 2014; Löwhagen Hendén, 2017; Mundiyanapurath, 2015; Nichols, 2018; Ren, 2020; Schönenberger, 2016; Simonsen, 2018; Sørensen, 2019; Zussman et al., 2018) No significant difference was found between general anesthesia and conscious sedation during the endovascular therapy for acute ischemic stroke. All the relationships had high p‐values suggesting that the increase in the number of studies included would not affect the level of significance if present. However, data stratified by the anesthetic strategy related to the symptomatic intracranial hemorrhage resulted in a very low *p*‐value (*p* = .05), almost significant, favoring general anesthesia suggesting that if one or more studies were included favoring general anesthesia the results could have been significant. Further studies are required to determine this relationship.

Former meta‐analyses suggested that general anesthesia compared to conscious sedation for endovascular therapy decreases recovery and increases death and disability. (Abou‐Chebl et al., 2010; Brinjikji et al., 2015; Wan et al., 2019) The link of general anesthesia with inferior results after endovascular therapy in observational studies (Abou‐Chebl et al., 2010, 2015; Just et al., 2016; Mundiyanapurath et al., 2015) may be clarified by blood pressure reductions and an extended delay of time to groin puncture in the general anesthesia group, both of which have been related to inferior endovascular therapy results. (Davis et al., 2012) Opposite to many nonrandomized studies and former meta‐analyses, this study appears to support the idea that endovascular therapy may be performed with both conscious sedation and general anesthesia with favoring general anesthesia due to its relatively lower symptomatic intracranial hemorrhage result (*p* = .05). The variance between our meta‐analysis study and former studies may be because general anesthesia was mostly selected for the subjects with a severe disability. This is confirmed by the higher average baseline National Institute of Health Stroke Scale scores for subjects having general anesthesia than those having conscious sedation in those studies. (Bekelis et al., 2017) Three large clinical trials showed similar results to ours. That would support the validity of our finding. (Löwhagen Hendén et al., 2017; Schönenberger et al., 2016; Simonsen et al., 2018) SIESTA (Sedation vs. Intubation for Endovascular Stroke Treatment), showed that general anesthesia versus conscious sedation did not result in a significant neurological status in subjects with ischemia undergoing endovascular thrombectomy. (Schönenberger et al., 2016) AnStroke (Anesthesia During Stroke), reported no difference between general anesthesia and conscious sedation in neurological outcome 3 month poststroke (*p* = 1.00). (Löwhagen Hendén et al., 2017) The last one, GOLIATH (General or Local Anesthesia in Intra Arterial Therapy), reported general anesthesia did not have inferior tissue or clinical results compared to conscious sedation. (Simonsen et al., 2018) Their finding supports our finding because of the large sample size they use compared to most other studies. (Löwhagen Hendén et al., 2017; Schönenberger et al., 2016; Simonsen et al., 2018).

A stratified analysis of studies that did and did not adjust for age and gender was not performed because not enough studies were reported or adjusted for this factor. However, from the study results presented here, we can recommend either anesthetic strategy during the endovascular therapy for acute ischemic stroke with no possible fear of higher complication.

Further studies are needed to evaluate the relationship of disease, subject, and management‐related variables to identify the best subject to undergo general anesthesia. Limits on age, conscious sedation (Glasgow Coma Scale), National Institute of Health Stroke Scale score, Alberta Stroke Program Early CT Score, and, time to treatment, need to be further studied. Furthermore, longer follow‐ups can help in providing more understanding of efficiency and safety. Lastly, cost‐effectiveness analyses should be followed to determine.

## 5 LIMITATIONS

The included articles were small in sample size, which has a potential risk of biases. There may be selection bias in this study since so many of the studies found were excluded from the meta‐analysis. However, the studies excluded did not satisfy the inclusion criteria of our meta‐analysis.

A stratified analysis of studies that did and did not adjust for age and gender was not performed because not enough studies were reported or adjusted for this factor.

All involved trials were in developed countries, and the management of general anesthesia is dependent more on advanced facilities and technical means than conscious sedation. Thus, these findings might not apply to developing countries. Further research in these countries would add to the finding. There was also some difference in methodology of the selected studies, for example, including criteria, and the type of instrument used for endovascular therapy, which might influence the results. All selected studies were not double‐blind.

## 6 CONCLUSIONS

Based on this meta‐analysis, opposite to most of the former meta‐analysis, conscious sedation has no independent relationship compared to general anesthesia during the endovascular therapy for acute ischemic stroke with a relative relationship favoring general anesthesia only in decreasing the symptomatic intracranial hemorrhage.

This relationship encouraged us to recommend either anesthetic strategy during the endovascular therapy for acute ischemic stroke with no possible fear of higher complication. Further studies are needed.

## CONFLICT OF INTEREST

The authors declare that they have no competing interests.

## Data Availability

The datasets analyzed during the current study are available from the corresponding author on reasonable request.
